# UV-Absorbing Ligand Capped Gold Nanoparticles for the SALDI-MS Analysis of Small Molecules

**DOI:** 10.5702/massspectrometry.A0107

**Published:** 2022-12-15

**Authors:** Tomomi Kakuta, Nichayanan Manyuan, Hideya Kawasaki

**Affiliations:** 1 Department of Chemistry and Materials Engineering, Faculty of Chemistry, Materials and Bioengineering, Kansai University, Osaka, Japan

**Keywords:** SALDI-MS, gold nanoparticles, UV-absorbing ligand, meropenem, small molecules

## Abstract

We report that modifying the surface of gold nanoparticles (Au NPs) with 2-mercaptopyridine-3-carboxylic acid (MPyCA) enhances surface-assisted laser desorption/ionization (SALDI) performance in the analysis of small molecules. The MPyCA ligand has a strong UV absorbance at the wavelengths of the typical MALDI laser at 337 nm, resulting in efficient thermal/energy transfer from the Au NPs to analytes during pulse-laser irradiation. In addition, the MPyCA ligand contains carboxylic acid and pyridine groups, providing affinity to various analytes through acid-base interactions.

Irganox1010, glucose and meropenem were utilized as model analytes to evaluate SALDI performance because these molecules are generally ionized with difficulty by conventional MALDI-MS. Our results demonstrate that the MPyCA-Au NP based SALDI-MS could detect Irganox1010, glucose and meropenem with stronger ion peaks for these molecules compared to MALDI-MS using CHCA. The limit of detection (LOD) for meropenem was much lower in the case of SALDI (LOD=1 ng/mL) compared to MALDI (LOD=10 μg/mL).

## INTRODUCTION

Matrix-Assisted Laser Desorption/Ionization (MALDI) is capable of the soft ionization of thermally unstable or high molecular weight molecules by laser irradiation and has been used in mass spectrometry for the analysis of high molecular weight molecules such as biopolymers and synthetic polymers.^1,2)^ In a typical MALDI experiment using an ultraviolet laser, an organic acid matrix with a strong UV absorption band is mixed with analytes to prepare a mixed crystal. The homogeneity of the matrix/analyte mixture affects the quality of the spectra, making optimization of the sample preparation a challenge. However, matrix-related ions and clusters thereof are detected together with the high-intensity analyte in the low mass region of the spectrum. Therefore, when the analyte is a small molecule of less than 700 Da, these ions may interfere with the mass spectral analysis, making the analysis a challenge. Many drugs, including medicines, plastic additives, and substances that cause environmental problems have molecular weights below 700. As a result, MALDI is rarely applied to such small molecules.

Surface-Assisted Laser Desorption/Ionization Mass Spectrometry (SALDI-MS) is a laser desorption/ionization mass spectrometry method in which an organic matrix is not used.^3)^ SALDI-MS involves the use of inorganic nanoparticles or substrates with nanostructured surfaces as desorption/ionization supports provides less interference with small matrix-derived molecular ions than the MALDI method.^4–6)^ This feature enables the sensitive detection of small organic molecules with weights below 700 Da. In addition, sample preparation is simple, enabling the simultaneous analysis of large quantities (high-throughput analysis), which would be expected to shorten analysis time. It has been proposed that “reaching a high temperature under rapid heating conditions” is one of the key factors for successful desorption/ionization without destroying molecules,^2,5,7)^ although non-thermal processes have also been proposed as possible mechanisms for desorption and ionization in SALDI-MS.^8,9)^ The desorption and ionization mechanisms in SALDI-MS continue to be a matter of debate.^10)^

A wide variety of nanomaterials including silicon, metal or metal oxide-based nanoparticles, metal-organic frameworks, polymers, and carbon-based materials have been reported to be effective materials for use in SALDI-MS as effective.^11–13)^ Their excellent optical absorption performance and superior thermal/charge transfer effectively improve the efficiency of laser desorption/ionization. Among these nanomaterials, gold nanoparticles (Au NPs) have been extensively reported for use in conjunction with SALDI-MS for a wide range of small molecules because of their unique optical properties that include surface plasmon resonance properties, temperature increase, and surface functionalization with a ligand for target analytes.^14–22)^ The surface chemistry and the size of Au NPs are crucial parameters for lowering the detection limits and increasing the selectivity of SALDI-MS. In some cases, gold-related ions that are observed in the mass spectra are a severe issue for Au NP-based SALDI-MS, since they interfere with the detection of low molecular weight analytes.^23,24)^ On the other hand, gold-related ions can be utilized for examining the distribution of Au NPs in a stained tissue section by imaging mass spectrometry.^25)^ Au NPs modified with **α-cyano-4-hydroxycinnamic acid (CHCA)** have been used for the analysis of peptides.^26)^ The CHCA surface modification suppressed the generation of gold-related ions in mass spectra and improved peptide ionization. However, combining CHCA-modified Au NPs with optimal amounts of glycerol and citric acid is still needed in order to improve the signal-to-noise ratio for peptide ions. In another approach, a mixture of Au NPs and low concentrations of CHCA was reported to enhance signals compared to conventional MALDI.^27)^ Thus, combining UV-absorbing molecules with Au NPs improves the analyte signals in mass spectra.

In the present study, we synthesized 2-mercaptopyridine-3-carboxylic acid (MPyCA) capped gold nanoparticles (MPyCA-Au NPs) for use in the SALDI-MS of small molecules. We used the MPyCA ligand for ligand-capped Au NPs of SALDI-MS for the following reasons. (1) the MPyCA ligand has a strong UV absorbance at the wavelengths of commercial MALDI lasers (337 nm) which would analyte signals in mass spectra compared to conventional MALDI. (2) The -SH group permits covalent bonds to be formed on the Au NP surface *via* the formation of Au–S bonds. (3) The MPyCA ligand contains carboxylic acid and pyridine groups, and these functional groups provide affinity for various analytes through acid-base interactions and hydrogen bonding. Herein, we examined the efficiency of MPyCA-Au NPs for use in the SALDI-MS analysis of glucose, meropenem sodium carbonate (carbapenem antibiotic), and Irganox® 1010 (antioxidant additives found in plastics). Our findings indicate that the performance of the MPyCA-Au NPs in SALDI-MS are superior compared to MALDI-MS using an organic matrix of CHCA for the detection of these small molecules.

## EXPERIMENTAL

### Materials

Hydrogen tetrachloroaurate(III) tetrahydrate (99.9%), hydrazine monohydrate (98.0%), sodium trifluoroacetate (97.0%), methanol (99.7%), tetrahydrofuran (99.5%) (THF), Irganox1010, polyethylene glycol 400 (PEG400), D(+)-glucose (98.0%), and meropenem sodium carbonate (>99.5%) were purchased from FUJIFILM Wako Pure Chemical Industries, Ltd. MPyCA (98.0%) and CHCA (99.0%) were purchased from Sigma Aldrich. The water used in this study was purified by a water distillation apparatus (water distillation apparatus, Aquarius RFD250, ADVANTEC).

### Synthesis of MPyCA-Au NPs

A 0.5 mL portion of a 0.2M HAuCl_4_ aqueous solution at pH 10 was added to 10 mL of an aqueous solution at pH 10 including 5 mg of MPyCA. The mixture was stirred at 1400 rpm for 1 h, and 1 mM N_2_H_4_ aqueous solution was then slowly added dropwise into solution A with stirring at 1400 rpm. The total volume of N_2_H_4_ aqueous solution added was 1.5 mL. The mixture was further stirred for 24 h.

### Purification of MPyCA-Au NPs for SALDI-MS

The as-prepared MPyCA-Au NP solution was purified for use in the SALDI-MS measurements to avoid contamination from by-products in the mass spectra. The as-prepared MPyCA-Au NP solution was adjusted to pH 3, resulting in the formation of a turbid solution. The sediments of MPyCA-Au NPs were collected by centrifugation at 24,145 g for 10 min, and the supernatant liquid was removed. The sediments were washed three times using 4 mL of water : methanol mixture (2 : 8 by volume). The precipitate was dried under reduced pressure for 24 h to obtain MPyCA-Au NPs as black solids. For the SALDI-MS measurement and the characterization, pure water was added to MPyCA-Au NP powder to produce a 1 mg/mL solution, and the pH was adjusted to around 10.0 to give a reddish brown solution.

### Characterization of MPyCA-Au NPs

The particle size of the MPyCA-Au NPs was determined by dynamic light scattering (DLS), using a particle characterization system (Zetasizer Nano ZSP. Malvern Panalytical, Malvern, UK, measurement range: 0.1–10000 nm, temperature: 25°C) that was equipped with a He–Ne laser (λ=633 nm) and back-scattering detector (173°). Transmission electron microscopic (TEM) images of the nanoparticles were captured at 120 kV on a JEOL 1400 microscope. The surface functional groups of the MPyCA-Au NP powders were characterized by FT-IR spectroscopy (FT/IR−4200, JASCO Corporation, Tokyo, Japan, measurement range: 500–4000 cm^−1^, resolution: 4 cm^−1^, number of scans: 128) with an attenuated total reflection (ATR) instrument (ATR PRO ONE, JASCO Corporation, Tokyo, Japan). The optical properties of the colloidal MPyCA-Au NPs were characterized by ultraviolet-visible (UV-vis) spectroscopy (V-670, JASCO Corporation, Tokyo, Japan).

### SALDI-MS measurements

The two-layer sample preparation method was employed for the SALDI-MS of small molecules with MPyCA-Au NPs:  the first step involved spotting the MPyCA-Au NPs solution (1 μL, 10 mg/mL), prepared by dispersing MPyCA-Au NPs in pure water at pH 10 with ultrasonication for 5 min on a stainless-steel plate, followed by drying; the second step was typically the deposition of a 1.0 μL sample solution on the plate. The amount of sample loaded in each experiment was a 1.0 μL solution in this study. Regarding glucose or meropenem, these substances were dissolved in a water/methanol mixture (1 : 1) to give a concentration of 1 mg/mL. To examine the detection limit for meropenem, meropenem solutions with various concentrations were also prepared. For Irganox 1010, THF was used as a solvent to produce a concentration of 1 mg/mL. MALDI-MS measurements were conducted using the dried droplet method with a CHCA matrix. Mass spectra were acquired in positive reflectron mode using a MALDI-TOF MS with a 337 nm laser (Bruker Microflex LRF; Laser Power 20%, Detector Gain 20 x, Method LP_0-2 kDa).

## RESULTS AND DISCUSSION

### Characterization of the MPyCA-Au NPs

**Figure 1:**
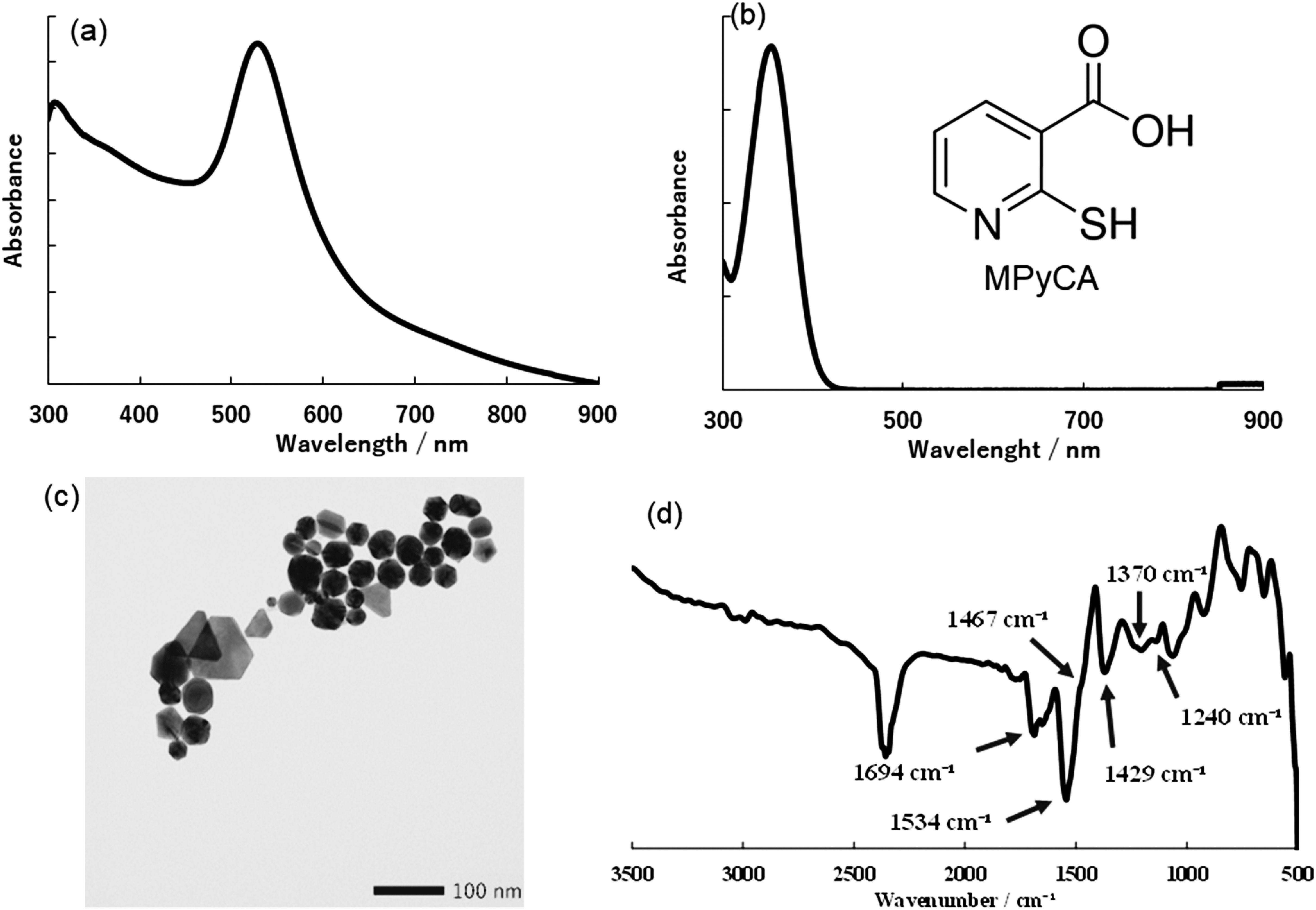
Fig. 1. (a) UV-Vis spectrum of 2-mercaptopyridine-3-carboxylic acid (MPyCA) Au NPs in water at pH 10. (b) UV-Vis spectrum of MPyCA in water at pH 10. (c) TEM image of MPyCA Au NPs. (d) FT-IR spectrum of MPyCA Au NPs.

Au NPs exhibit a strong absorbance band in the visible region (500–600 nm), commonly referred to as localized surface plasmon resonance (LSPR), that is, the collective oscillation of electrons in the conduction band of the Au NPs in resonance with a specific wavelength of incident light, which can be measured by UV-Vis spectroscopy.^28)^ To serve as the matrix of SALDI-MS, the first priority of the MPyCA-Au NP is the absorption capability of the laser at 337 nm. The colloidal MPyCA-Au NPs were measured by UV-Vis absorption spectroscopy. As shown in Fig. 1a, the MPyCA-Au NPs show a strong LSPR absorbance at around 550 nm, indicating the formation of Au NPs. More importantly, the MPyCA-Au NPs exhibit a very high absorption at 337 nm as well as the unattached MPyCA (Fig. 1b) . Thus, the MPyCA-Au NPs meet the requirements to serve as matrices in SALDI-MS.

The Z average particle size and polydispersity index (PDI) of the MPyCA-Au NPs in water at pH 10 were estimated by DLS, and found to be 70.8 nm and 0.422, respectively. A PDI value of more than 0.1 indicates polydisperse particle size distributions. The TEM images of the MPyCA-Au NPs showed polydisperse particle size distributions of less than 100 nm, and irregular shaped Au NPs were observed in addition to spherical NPs (Fig. 1c).

Figure 1d shows the FT-IR spectrum of MPyCA-Au NPs, supporting the surface modification of Au NPs by MPyCA based on the following assignments. Stretching vibrations of C-N at 1240 cm^−1^; a C=C bond stretching vibration at 1467 cm^−1^; two COO- bond stretching vibration peaks at 1534 cm^−1^ and 1429 cm^−1^.

### MPyCA-Au NP-based SALDI-MS of small molecules

**Figure 2:**
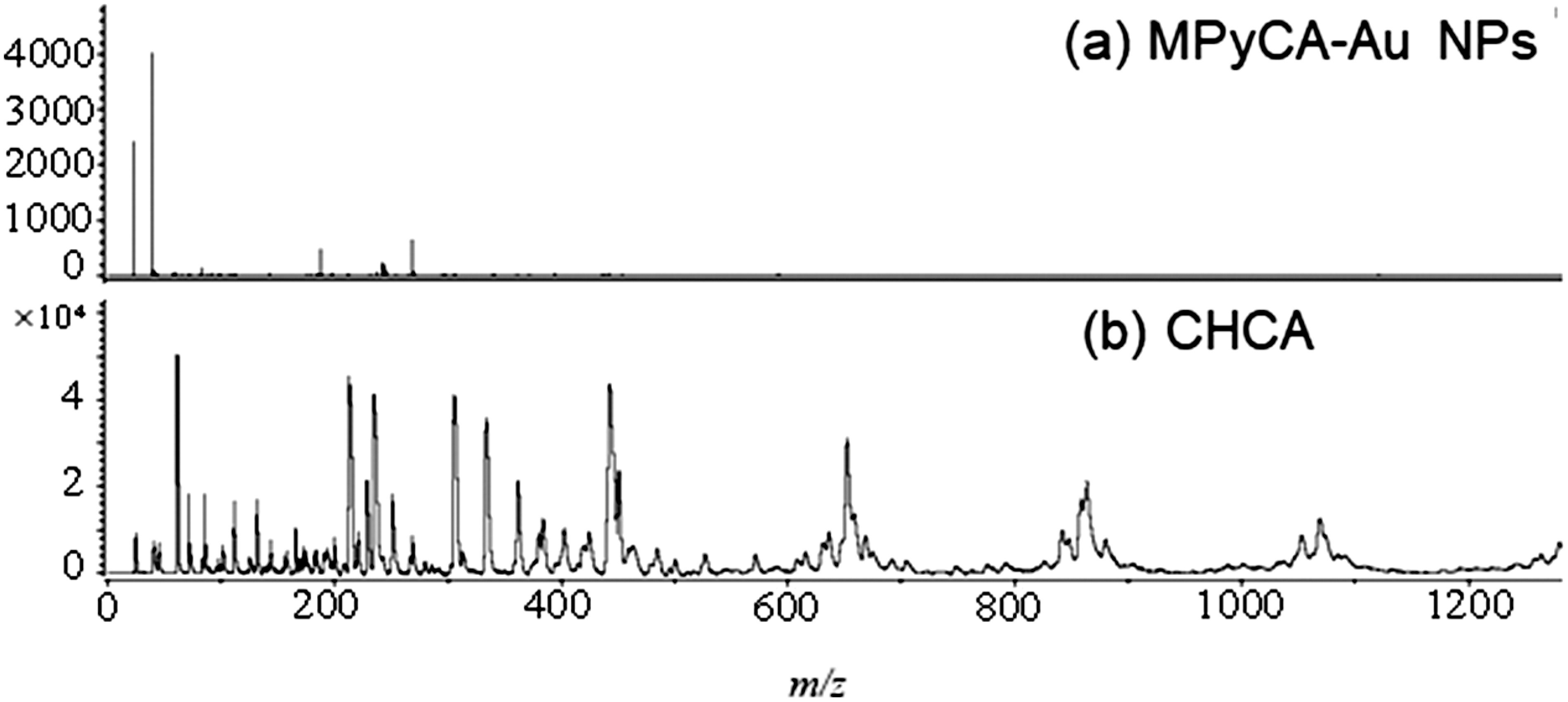
Fig. 2. LDI mass spectra of (a) MPyCA-Au NPs and (b) CHCA.

To utilize MPyCA-Au NP-based SALDI-MS for the analysis of small molecules, background signals derived from the NPs in the low-mass region should be avoided. Background signals can usually arise from two major sources: unreacted ligands and solvent residues. As described above, the unreacted residual ligands can be removed by repeated washing with a water/methanol mixture after the synthesis. We first compared the matrix-related ions of SALDI-MS using MPyCA-Au NPs and MALDI-MS using CHCA. Figure 2 shows LDI mass spectra of (a) MPyCA-Au NPs and (b) CHCA. Only a few peaks from the MPyCA-Au NPs were observed in the mass spectrum, while CHCA showed abundant matrix-related ions.

**Figure 3:**
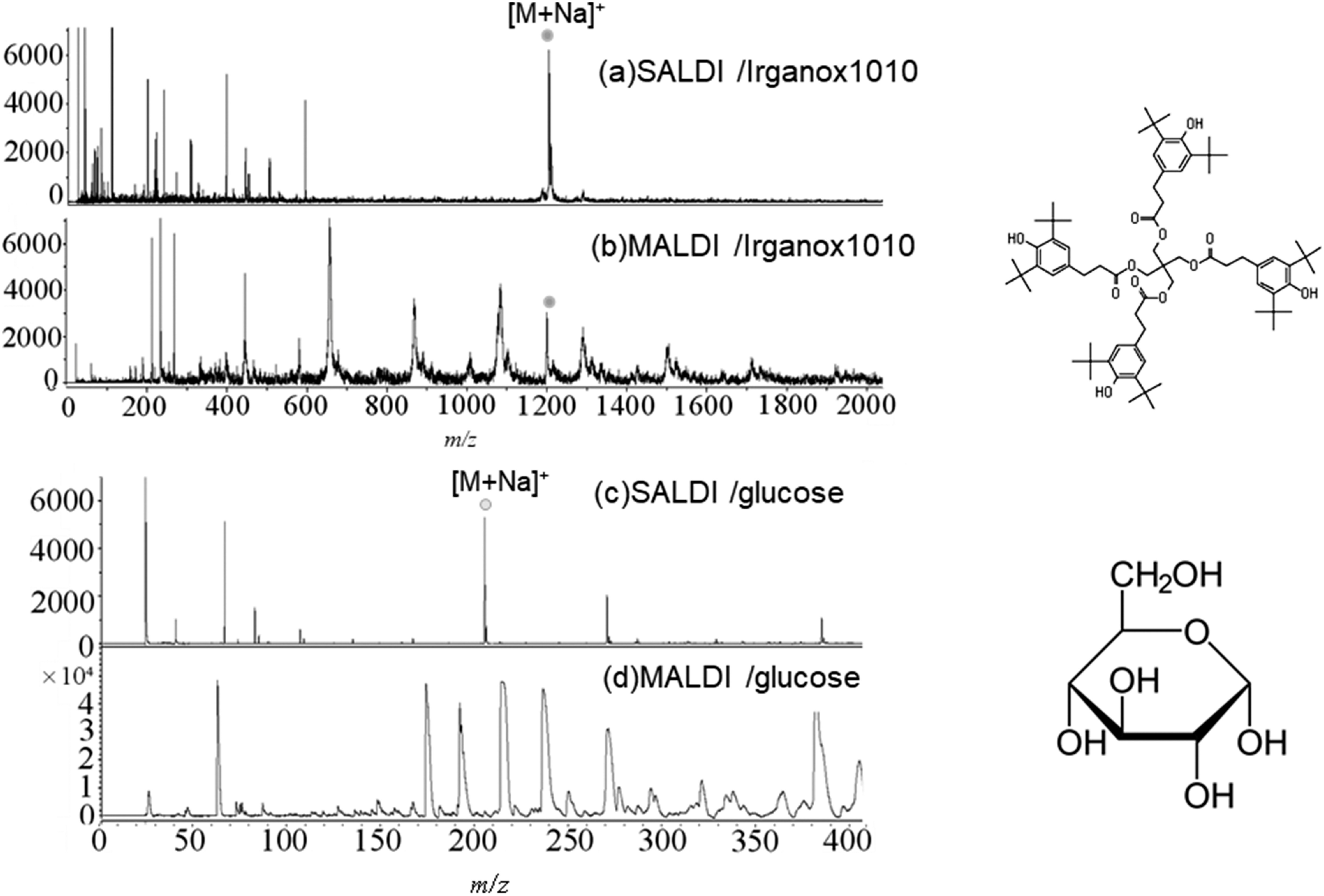
Fig. 3. SALDI and MALDI mass spectra of (a), (b) Irganox1010 and (c), (d) glucose.

Several molecules, including an antioxidant Irganox1010 (Ciba Japan K.K., Tokyo, Japan) and glucose, were employed as test samples. The carboxylic acid and pyridine groups of MPyCA-Au NPs provide affinity for the hydroxy groups of glucose and Irganox 1010 through acid-base interactions and hydrogen bonding.^29)^ Figure 3 shows SALDI and MALDI mass spectra of (a), (b) Irganox1010 and (c), (d) glucose. The ion peak at *m*/*z* 1200 is observed as a sodium adduct [M+ Na]^+^ of Irganox 1010 in the SALDI and MALDI mass spectra. The background noise was higher in the case of MALDI compared to SALDI. Several peaks in the low mass region in the SALDI are not assigned, and these may be due to contaminants in the Irganox 1010.

Glucose is often utilized to evaluate the capability of SALDI-MS for small molecules. The MPyCA-Au NP-based SALDI allowed the detection of glucose in the mass spectrum as the sodium adduct [M+Na]^+^ ion at *m*/*z* 203 with a high signal intensity (Fig. 3c). As shown in Fig. 3d, the interferences arising from the CHCA matrix was substantial for glucose detection in the case of MALDI-MS, but there were no signals for glucose in the MALDI-MS. These results demonstrate that MPyCA-Au NP-based SALDI has great potential for use in detecting small molecules by SALDI-MS.

### Detection of an antimicrobial agent

To further expand the applicable range of the MPyCA-Au NP-based SALDI, we examined the detection of meropenem (an antimicrobial agent) by using MPyCA-Au NPs-based SALDI-MS and CHCA-based MALDI-MS. Meropenem is a carbapenem antibiotic that is effective against many pathogenic bacteria (influenza, *Escherichia coli*, Streptococcus pneumonia, *etc.*).^30)^ The number of bacteria that are resistant to antimicrobial agents (carbapenemases) has been increasing. More and more patients are suffering from severe diseases due to the ineffectiveness of these agents as they try to survive exposure to antimicrobials.^31)^ When the antimicrobial agent comes into contact bacteria, the molecular weight of the antimicrobial agent changes as the result of the hydrolysis of the β-lactam ring in the agent.^32)^ If SALDI-MS could be used to measure the molecular weight of the structurally altered antimicrobial, it would be possible to select an appropriate antimicrobial agent that is not affected by this bacterial strain. Monitoring the metabolites derived from meropenem using the SALDI-MS could be used to accomplish this. However, the use of SALDI-MS for the detection of meropenem has not been reported.

**Figure 4:**
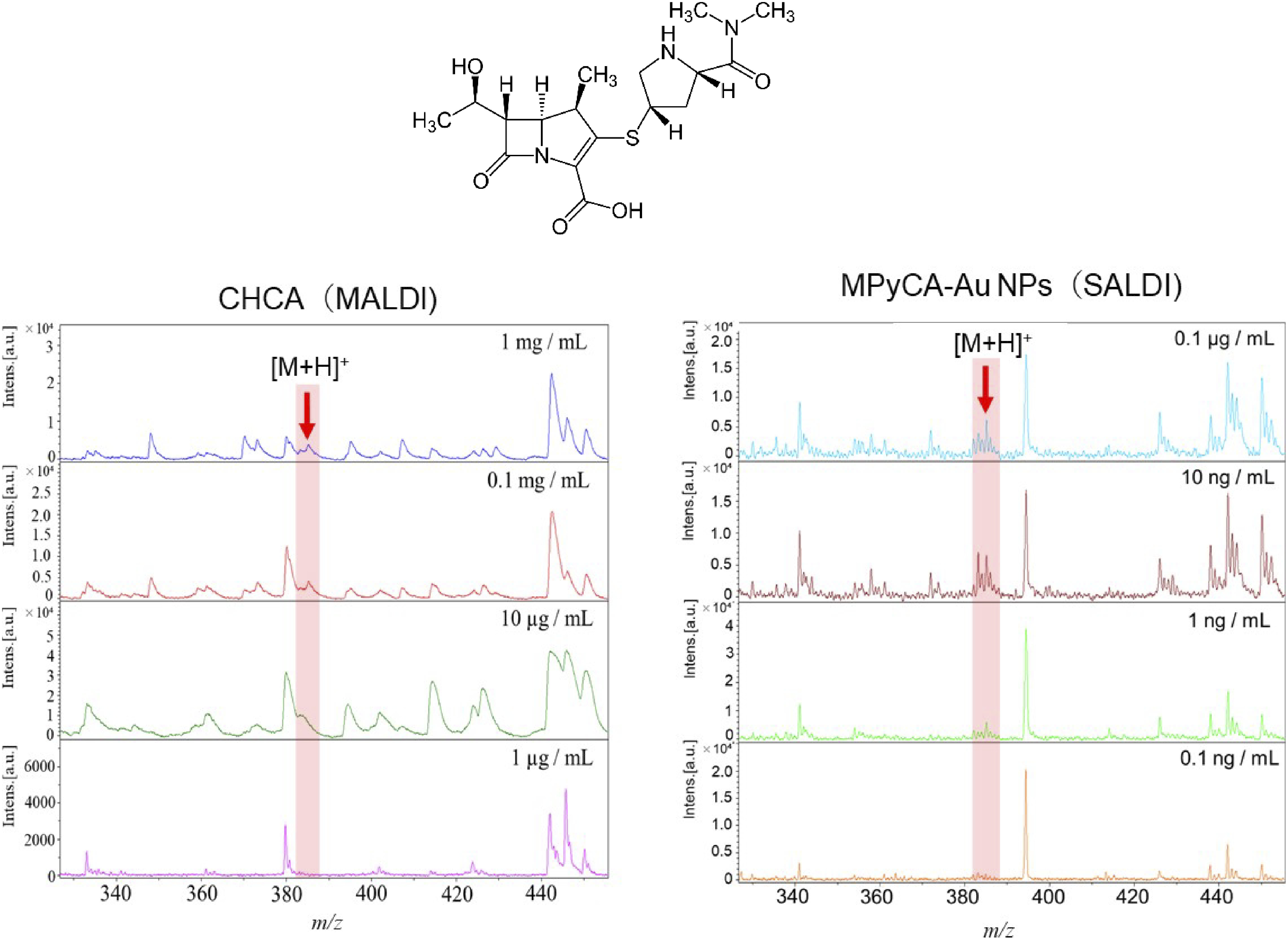
Fig. 4. SALDI and MALDI mass spectra of meropenem. The analyte concentrations are described in the figures. The chemical structure of meropenem is also shown in the figure. 1.0 μL sample solutions were used in the measurements.

Figure 4 shows SALDI and MALDI mass spectra of meropenem. The MALDI-MS obtained using CHCA can detect a protonated ion of meropenem at *m*/*z* 384 in the mass spectra from analyte solutions of 1 mg/mL, 0.1 mg/mL, and 10 μg/mL, but not 1 μg/mL. The detection limit of meropenem is around 10 μg/mL. In contrast, SALDI-MS using MPyCA-Au NPs showed a much higher sensitivity for the detection of the protonated ion derived from meropenem. The limit of detection of meropenem is about 1 ng/mL (Fig. 4). Therefore, MPyCA-Au NPs are promising candidates for use in high-sensitivity SALDI to detect meropenem as well as other analogous low-molecular-weight molecules. The MPyCA ligand on the NP surface may interact with meropenem *via* acid-base interactions, since both MPyCA and the meropenem molecule contain acid-base functional groups (carboxylic acid as an acidic group and pyridine/amino as a basic group), resulting in the meropenem molecule being adsorbed on the surface of the MPyCA-Au NPs. In SALDI-MS, the desorption/ionization process can also involve thermal effects due to the rapid temperature increase of the nanoparticle’s surface during laser irradiation.^2,5)^ Therefore, it appears likely that efficient thermal/energy transfer from the Au NPs to the molecules adsorbed to the surface occurs during the pulse-laser irradiation.

## CONCLUSION

We synthesized a new type of MPyCA capped Au NPs for use in SALDI-MS. The MPyCA ligand has a strong UV absorbance at the wavelengths of commercial MALDI lasers at 337 nm, resulting in the efficient transfer of energy from the AuNPs to analytes during pulse-laser irradiation. Because of the high stability of MPyCA-Au NPs, no interfering Au ion peaks from the NPs were observed in the SALDI-MS spectra. The SALDI-MS of Irganox1010, glucose and meropenem were investigated using MPyCA-Au NPs because these molecules are generally difficult to ionize by conventional MALDI-MS. The MPyCA-Au NP-based SALDI-MS showed stronger ion peaks for these molecules compared to the case of MALDI-MS using CHCA. In particular, the limit of detection for meropenem was found to be much lower for SALDI than for MALDI. The MPyCA-Au NP-based SALDI-MS presented in the contribution the potential to be applied to other small molecules of interest in the biological and environmental sciences.

## Notes

Mass Spectrom (Tokyo) 2022; 11(1): A0107
